# Cancer Treatment and Bone Health

**DOI:** 10.1007/s00223-017-0369-x

**Published:** 2018-01-20

**Authors:** Catherine Handforth, Stella D’Oronzo, Robert Coleman, Janet Brown

**Affiliations:** Department of Oncology and Metabolism, Academic Unit of Clinical Oncology, Weston Park Hospital, University of Sheffield, Sheffield, UK

**Keywords:** Cancer treatment, Osteoporosis, BMD, Prostate cancer, Breast cancer

## Abstract

Considerable advances in oncology over recent decades have led to improved survival, while raising concerns about long-term consequences of anticancer treatments. In patients with breast or prostate malignancies, bone health is a major issue due to the high risk of bone metastases and the frequent prolonged use of hormone therapies that alter physiological bone turnover, leading to increased fracture risk. Thus, the onset of cancer treatment-induced bone loss (CTIBL) should be considered by clinicians and recent guidelines should be routinely applied to these patients. In particular, baseline and periodic follow-up evaluations of bone health parameters enable the identification of patients at high risk of osteoporosis and fractures, which can be prevented by the use of bone-targeting agents (BTAs), calcium and vitamin D supplementation and modifications of lifestyle. This review will focus upon the pathophysiology of breast and prostate cancer treatment-induced bone loss and the most recent evidence about effective preventive and therapeutic strategies.

## Introduction

Advances in systemic cancer therapies and diagnostic techniques have led to consistent improvements in cancer survival, and, as a result, the long-term sequelae of cancer treatment have become an increasingly important consideration. Treatment-related adverse effects significantly impact the quality of life of patients, many of whom will live for years following their cancer diagnosis. They are also associated with significant economic costs to health and social care providers.

Bone health is an area of particular importance for patients with breast and prostate tumours. These are the commonest malignancies to affect females and males, respectively. More than 460,000 women are diagnosed with breast cancer (BC) in Europe each year, and BC is responsible for 130,000 deaths [1]. More than 420,000 men are diagnosed with prostate cancer (PC) in Europe each year, with more than half of cases occurring in men aged over 70 years [2]. Survival rates from both BC and PC have significantly improved over the past 40 years as a result of both treatment advances and earlier diagnosis, due to improved diagnostic techniques and screening campaigns.

In BC and PC, both the disease itself and the treatments pose specific challenges to skeletal integrity. This review will focus upon bone loss that occurs as a direct result of cancer therapy (cancer treatment-induced bone loss, CTIBL) for BC and PC. We will describe the factors that regulate bone remodelling processes, the pathophysiology and clinical complications of CTIBL. Evidence to support the use of bone-targeted agents (BTAs) will also be discussed, along with current guidelines for CTIBL management.

## Influence of Sex Steroids on Bone

Both androgens and oestrogens have important roles in bone growth and maturation, and maintenance of skeletal integrity. However, accumulating evidence suggests a role for other reproductive hormones, such as activins and inhibins, in the preservation of bone health.

### Androgens

Testosterone is the most abundant circulating androgen in men, 95% of which is secreted by the testis. The remaining 5% is formed from the enzymatic conversion of adrenal androgens dehydroepiandrosterone (DHEA) and DHEA sulphate (DHEAS) [3]. In women, the major circulating androgens are produced by the adrenal glands and ovaries and include DHEAS and DHEA, androstenedione (pro-androgens), testosterone and dihydrotestosterone (DHT). Female testosterone production also occurs via peripheral aromatisation of androstenedione. In both men and women, the majority of circulating testosterone is protein bound (to either sex hormone-binding globulin or albumin).

Testosterone may act directly on androgen receptors (AR), or indirectly via aromatisation to oestradiol and activation of oestrogen receptors (ER). The indirect actions of testosterone may also occur following conversion to the more potent DHT by 5α-reductase in peripheral tissues [4].

In bone, androgens exert direct effects on growth plate chondrocytes and promote longitudinal bone growth [5]. Both testosterone and DHT stimulate the proliferation of osteoblast precursors via AR signalling [6]. Binding of androgens with AR also upregulates osteoblast AR expression and promotes their differentiation [7]. Androgens also prevent osteoblast and osteocyte apoptosis and regulate osteoclast activity by inhibiting the interaction of receptor activator of nuclear factor kappa-B ligand (RANK-L) with its receptor (RANK), expressed on osteoclast precursors [8, 9]. Bone formation is promoted by androgen-mediated upregulation of growth factors, such as insulin-like growth factor and transforming growth factor beta, TGFβ [6]; on the other hand, downregulation of interleukin 6 inhibits osteoclast activity [10] through the reduction in levels of osteoprotegerin (OPG), which is produced by osteoblasts and acts as a soluble decoy receptor to RANK-L [11].

### Oestrogens

The net action of oestrogens on bone is to decrease bone resorption. Their actions are exerted via ERα and ERβ receptors, expressed by osteoblasts and osteoclasts. Oestrogens increase osteoblast number and activity, inhibit the maturation of osteoclast precursors (via increased OPG production) and also cause osteoclast apoptosis [12, 13]. Oestrogen deficiency is known to increase the rate of osteocyte apoptosis with a consequent increase in skeletal fragility [14, 15].

In women, oestrogens are critical for the maintenance of normal bone mass. At the menopause, loss of ovarian follicular activity causes a significant fall in circulating oestrogens, with a consequent disruption of bone remodelling. The most rapid bone loss occurs in the first 3 years post-menopause (2–5%/year), after which skeletal metabolism becomes “acclimatised” to the low-oestrogen environment and bone loss slows to around 0.5–1.0% per annum. A greater proportion of bone loss occurs at sites containing trabecular bone (such as the spine) than cortical sites (such as the hip) [16].

Oestrogens have been identified as the sex steroids primarily responsible also for the regulation of bone resorption in men. In studies of young male patients unable to produce or respond to oestrogens, there was an increased rate of bone turnover and osteopaenia [17, 18]. Both oestrogens and testosterone are known to be important in the regulation of male bone formation [19].

### Activins and Inhibins

Activins and inhibins belong to the TGFβ superfamily and exert their biological activity by interacting with a type II serine/threonine kinase receptor; once the latter associates with the corresponding type I receptor, an intracellular pathway is activated, leading to the regulation of DNA transcription, mediated by Smad proteins [20].

Activin A and B are homodimeric peptides, made up of βA and βB subunits, respectively, and regulated by gonadotropins from the pituitary. Activin A is produced in the gonad, while activin B is secreted by the pituitary gland itself; in the circulation, they are bound to follistatin in order to prevent excessive interaction with the activin type II receptor (ACTRII). Interestingly, neither activin A nor follistatin serum levels vary according to the menopausal status [21]. BC cells have been shown to produce activin, which in turn inhibits the proliferation of ER + tumour cell lines [22]; however, its role in the establishment of bone metastases has not been clarified yet. Moreover, while its capability to promote osteoclastogenesis via RANK upregulation has been ascertained, conflicting data exist about the role of activin in osteoblastogenesis [21].

Inhibin A and B are heterodimeric proteins, including αβA or αβB subunits. Due to their capability to prevent the interaction of activin with ACTRII, inhibins suppress gonadotropin secretion from the pituitary gland. Inhibin B levels tend to decline with age in both women and men, thus representing a good marker for fertility [23]. Similarly to activins, inhibins are secreted by BC cells [24] although their role in carcinogenesis seems contradictory. As activin inhibitors, one would expect them to promote tumour cell proliferation, but in vivo and clinical data suggest a possible tumour-suppressive activity [22]. With respect to bone turnover, this seems to be counteracted by inhibins through the suppression of both osteoblastogenesis and osteoclastogenesis [21]. Moreover, in a cross-sectional study involving 188 women with different ages (from 21 to 85 years), inhibins turned out as the best predictors of bone marker fluctuations, potentially able to indicate the initial bone loss that characterises the perimenopausal period, even before sex steroid and gonadotropin variations could be detected [25].

## Bone Loss in Prostate Cancer

### Pathophysiology

The growth of PC is initially androgen dependant, and the cornerstone of treatment is androgen deprivation therapy (ADT). This is most often achieved by the administration of luteinising hormone-releasing hormone (LHRH) agonists, LHRH antagonists and antiandrogens. ADT provides excellent initial disease control in virtually all patients; however, many of them subsequently develop castration resistance. Following the initiation of ADT, sex steroid levels decrease rapidly and significantly, reaching a nadir within 2–4 weeks (less than 5 and 20% of normal testosterone and oestrogen levels, respectively). This leads to an accelerated and disorganised bone turnover process and net bone loss that is most rapid during the first year of ADT (studies have reported between 5 and 10% loss of bone mineral density, BMD) [26–28]. BMD loss also continues gradually throughout ADT duration. In a study of 390 men aged 54–89, the prevalence of osteoporosis was found to be 35% in hormone-naïve patients, 43% after 2 years of ADT and 81% after 10 years of ADT [29].

The pattern of CTIBL associated with ADT has been shown to differ from that seen with other conditions, being more likely to affect the radius than the hip or spine [30]. However, recent evidence suggests that this phenomenon could be partially attributed to the frequent overestimation of central BMD in men with skeletal degenerative disorders, which commonly occur in the elderly. On the other hand, densitometers have been found to over-read radius osteoporosis when using a male normative database, and thus forearm BMD should be re-analysed and evaluated according to larger normative databases [31].

In addition to ADT, men with newly diagnosed hormone-sensitive metastatic PC may receive chemotherapy [32]. Docetaxel is given for 18 weeks, along with daily 10 mg prednisolone and 24 mg dexamethasone every 3 weeks. It is well established that long-term use of glucocorticoids is associated with osteoporosis; indeed, it is the most common cause of secondary osteoporosis. Evidence suggests that the risk of hip and vertebral fractures increases up to 7- and 17-fold, respectively, when doses equivalent to 10–12 mg prednisolone are given for more than 3 months. The risk of fracture is also increased even when small (2.5–3 mg) daily doses are given [33]. No studies to date have investigated the impact of the combination of chemotherapy and glucocorticoids with ADT on BMD and fracture risk in men with PC.

### Clinical Sequelae

CTIBL that is associated with ADT exponentially increases the risk of fracture. Indeed, the loss of 10–15% of BMD doubles the fracture risk [34] and men receiving ADT for PC are five times more likely to develop a fracture than healthy age-matched controls [35].

In particular, Shahinian et al. retrospectively evaluated the fracture risk of 50,613 PC patients listed in the Surveillance Epidemiology and End Results (SEER) programme between 1992 and 1997. Among men who survived at least 5 years after PC diagnosis, those receiving ADT exhibited a significantly higher risk of fracture, as compared to untreated men (19.4 vs. 12.6%, *p* < 0.001). Moreover, patients undergoing orchiectomy or receiving at least nine doses of LHRH agonists had the lowest fracture-free survival, although the analysis did not exclude bone metastasis-related fractures [36].

Another similar analysis involved 11,661 patients with non-metastatic PC and confirmed the significantly higher fracture rate in men undergoing ADT (7.88 vs. 6.51%/year of controls, *p* < 0.001), and the highest hazard ratio (HR) in those receiving LHRH agonists for at least 12 months (1.16, 95% CI 1.08–1.26; *p* < 0.001). Interestingly, when considering fractures at specific sites, both vertebral and hip/femur ones were more frequent in patients undergoing ADT, as compared to controls (*p* < 0.001 and *p* = 0.002, respectively) [37].

Not only are such events associated with subsequent fractures and loss of independence, but they also represent an independent adverse predictor of survival. Indeed, the relative risk of death is sevenfold greater in men with PC receiving ADT and who have a previous fracture compared to those with no fracture history [38].

## Bone Loss in BC

Since the early 1990s, although the incidence of BC has steadily risen, BC mortality has progressively decreased. This “good news” has, however, raised concerns about the possible long-term effects of cancer treatments. In particular, both chemotherapy and hormone treatment have an impact on bone health, with different mechanisms that will be discussed in the next sections.

### Pathophysiology of Chemotherapy-Induced Bone Loss and Clinical Data

Adjuvant chemotherapy has a significant impact on bone health in BC, due to both the induction of premature menopause and direct effects on bone turnover [39]. For example, cyclophosphamide exerts gonadotoxic effects (the metabolite phosphoramide mustard damages at first granulosa cells and then oocytes in a dose-dependent manner) [40] and also affects cell division of both osteoclast and osteoblast precursors, thus interfering with physiological bone turnover [41]. Additionally, doxorubicin, although less gonadotoxic, can also induce both premature ovarian failure in vivo [42] and increased osteoclast differentiation in vitro, at the expense of osteoblasts [43].

Studies investigating the effects of adjuvant chemotherapy on BMD in women with early BC have all reported a decrease in lumbar spine (LS) BMD, as compared to baseline [44–47]. In particular, Shapiro and coworkers investigated the effects of adjuvant chemotherapy (containing cyclophosphamide and/or doxorubicin) on the bone health of 49 premenopausal patients with early BC; interestingly, all women experiencing premature ovarian failure (35/49) showed a significant BMD reduction at the LS within the first 6 months (− 4.0%, *p* = 0.0001); this was greater than the bone loss seen in women who retained menses (− 1.0%) [44].

In another study conducted by Cameron et al., 41 premenopausal patients received adjuvant chemotherapy for early BC and were monitored in their bone health and ovarian function for 1 year. During the first 6 months, all patients experienced LS BMD decrease (*p* < 0.0001), independently of age, ovarian function and type of chemotherapy; during the following 6 months, LS BMD further decreased only in women experiencing amenorrhea and/or low estradiol levels (*p* < 0.0001) [47].

A recent prospective study reported BMD changes in 97 women (mean age: 53 years) with early BC who had completed anthracycline- or taxane-based adjuvant chemotherapy [48]. All patients received high-dose prednisolone as antiemetic prophylaxis and supplementation with calcium and vitamin D. At completion of chemotherapy, BMD at LS increased significantly in postmenopausal women compared to baseline (+ 2.35%, 95% CI 1.6–3.3, *p* < 0.001), whereas the BMD of premenopausal women did not change. Such effects could be partially explained by the vitamin supplementation which could prevent the typical calcium deficiency occurring in postmenopausal women.

Based on these data, chemotherapy-induced bone loss is currently interpreted more as the consequence of premature ovarian failure, rather than the result of direct bone damage.

### Pathophysiology of Hormone Treatment-Induced Bone Loss and Clinical Data

#### Long-Acting Gonadotropin-Releasing Hormone Agonists

Long-acting gonadotropin-releasing hormone (GnRH) agonists may be administered to premenopausal individuals with hormone receptor-positive (HR+) BC. Persistent activation of GnRH receptors leads to their desensitisation and consequent suppression of ovarian function. Induction of hypogonadism in premenopausal women is associated with accelerated bone loss which is usually, at least in part, reversible once treatment is stopped, especially in those who resume menses [49].

The Zoladex Early Breast Cancer Research Association (ZEBRA) bone sub-study included 96 women with premenopausal node-positive BC who had been randomised to receive either goserelin or CMF chemotherapy (cyclophosphamide, methotrexate and 5-fluorouracil). After 2 years of hormone treatment, goserelin was associated with a significantly greater reduction in BMD at both LS (mean BMD loss − 10.5 vs. − 6.5%, *p* = 0.0005) and femoral neck (FN) (− 6.4 vs. − 4.5%, *p* = 0.04), as compared to the CMF group. After a 3-year follow-up, partial BMD recovery was observed in goserelin-treated patients, but this did not occur in the CMF group [50].

However, the strongest evidence of goserelin impact on bone health derives from the Austrian Breast and Colorectal Cancer Study Group trial-12 (ABCSG-12), in which 404 premenopausal women with BC were randomised to receive adjuvant hormone treatment with or without zoledronate. Endocrine therapy consisted of either goserelin + tamoxifen or goserelin + anastrozole combination and, once administered without the bisphosphonate (BP), was associated with significant BMD reduction, evaluated after 36 and 60 months and compared to baseline values (after 36 months: LS BMD − 11.3%, FN BMD − 7.3%, *p* < 0.0001 in both instances; after 60 months: LS BMD − 6.3%, FN BMD − 4.1%, *p* = 0.0001 and *p* = 0.058, respectively). On the other hand, women receiving zoledronate exhibited stable and increased BMD after 36 and 60 months, respectively [51].

#### Tamoxifen

Tamoxifen is a selective oestrogen receptor modulator (SERM) exerting both antagonist (breast) and partial agonist effects (bone and uterus), that is widely used as treatment for women with HR + BC and as chemoprevention in women at high risk for developing BC. A placebo-controlled BC chemoprevention trial investigated the effects of tamoxifen on bone health in 179 women. In premenopausal women treated with tamoxifen, there was a mean annual BMD loss of 1.44% at LS, compared with a gain of 0.24% in the placebo group (*p* < 0.001). Tamoxifen had the opposite effect in postmenopausal patients, in whom it was associated with increased BMD at the LS (mean annual increase of 1.17%) compared with placebo (insignificant BMD loss) [52]. Subsequent studies involving postmenopausal women treated with tamoxifen reported reductions in bone turnover markers (BTM) [53] and significantly increased LS trabecular bone score (TBS), a novel parameter which reflects bone microarchitecture and is associated with resistance to fractures [54].

#### Aromatase Inhibitors

Third-generation aromatase inhibitors (AIs) induce a rapid decrease in circulating estradiol levels by preventing the conversion of androgens to oestrogens. Therefore, they represent a standard adjuvant treatment for postmenopausal patients with HR+ BC. Steroidal AIs (e.g. exemestane) irreversibly bind to the aromatase enzyme, while non-steroidal AIs (e.g. anastrozole, letrozole) are competitive inhibitors which bind to the heme moiety of the aromatase cytochrome P450 complex [55]. Table 1 summarises the most important studies investigating the effects of AIs on bone health.

**Table 1:** Clinical trials investigating the effects of aromatase inhibitors on fracture incidence

Clinical trial	Number of patients	Experimental arm	Control arm	Overall/annual fracture incidence	References
ATAC	9366	Anastrozole	Tamoxifen	Overall: 11 vs. 7.7% Annual: 2.93 vs. 1.90%	[57, 58]
IBIS II bone sub-study	1410	Anastrozole	Placebo	Annual: 1.37 vs. 1.26%	[100]
BIG 1.98	4895	Letrozole	Tamoxifen	Overall: 9.3 vs. 6.5% Annual: 2.71 vs. 1.872%	[59]
MA-17	5187	Letrozole	Placebo	Overall: 3.6 vs. 2.9%	[60]
IES	4274	Exemestane	Tamoxifen	Overall: 7 vs. 5% Annual: 2.01 vs. 1.60%	[61]
ABCSG-18	3420	AI + denosumab	AI + placebo	Overall: 5 vs. 9.6%	[107]

*AI* aromatase inhibitor

The ATAC trial investigated the effects of adjuvant anastrozole on bone health [56]. The study reported an increased fracture risk in patients treated with anastrozole, as compared to tamoxifen (2.93 vs. 1.90% annual fracture rate, *p* < 0.0001). However, after the treatment was suspended, anastrozole-treated patients experienced BMD recovery at LS and no further loss at the hip, suggesting that AI-induced bone loss is reversible [57, 58].

Adjuvant letrozole has been found to be associated with an increase in annual (2.71 vs. 1.87%) and overall fractures (9.3 vs. 6.5%) when compared with tamoxifen [59]. The MA17 trial included over 5000 women and confirmed that the addition of letrozole, after standard 5-year adjuvant therapy with tamoxifen, was associated with significantly improved disease-free survival (DFS) in postmenopausal women [60]. However, as compared to placebo, letrozole was also associated with a slightly increased incidence of osteoporosis (5.8 vs. 4.5%, *p* = 0.07), although the rates of fracture were similar in the two treatment groups.

In the Intergroup Exemestane Study (IES), postmenopausal women completing 2–3 years of adjuvant tamoxifen were randomised to continue the treatment until the fifth year or switch to exemestane. Those in the exemestane arm experienced a greater decrease in BMD and, in the intention-to-treat population, higher annual fracture incidence than those who continued to take tamoxifen (2.01 vs. 1.60% with tamoxifen) [61]. Fracture rates associated with the use of non-steroidal and steroidal AIs appear to be similar [62].

A recent retrospective study evaluated LS BMD and TBS variations in 321 non-osteoporotic patients treated with AIs for at least 3 years. Both parameters significantly decreased from baseline to 5 years (− 6.15 and − 2.12%, respectively; *p* < 0.001 in both instances) although the annual reduction tended to slow after 3 and 1 years of treatment, respectively [63].

An interesting line of current research aims to investigate CYP19A1 (aromatase) gene polymorphisms potentially associated with higher susceptibility to AI-induced bone loss. It has been reported that G-to-A substitution at Val80 in the exon 3 (rs700518) is apparently related to significant BMD decrease at LS and hip at 12 months [64]. More recently, a significant correlation between another polymorphism (rs4646, GG genotype) and osteoporosis during AI treatment has been described [65].

## Guidelines for Bone Health Assessment

### Prostate Cancer

European guidelines for PC were published jointly by the European Association of Urology (EAU), International Society of Geriatric Oncology (SIOG) and European Society for Radiotherapy and Oncology (ESTRO) in 2016 [66]. These recommend that all men starting long-term ADT should undergo dual-energy X-ray absorptiometry (DXA) assessment and the result used in conjunction with the FRAX^®^ tool to evaluate individual fracture risk. The FRAX^®^ tool is based upon data from prospectively studied population cohorts [67]. This tool is available online and may be used for individuals aged 40–90 years across multiple countries and regions of the world to predict the 10-year risk of hip and major osteoporotic fractures, on the basis of multiple risk factors (e.g. age, sex, smoking, alcohol intake, history of fractures, familiar history of hip fractures, body mass index < 20 kg/m^2^, long-term use of corticosteroids, comorbidities, BMD).

However, these guidelines lack specific recommendations regarding the ongoing monitoring and follow-up of CTIBL. Previous EAU guidelines suggested that the initial T score should guide the frequency of BMD measurement at the time of ADT initiation (DXA to be repeated annually if the T score is between − 2.5 and − 1.0 at baseline, or every 2 years if the initial T score is above − 1.0) [68]. The International Society for Clinical Densitometry suggests that BMD measurement should be repeated after the first year of ADT, which seems appropriate given that the most rapid decline in BMD is observed during the first year of therapy [69].

Despite available guidelines, the timing and frequency of DXA assessments in men with PC are subject to significant variation and depend upon clinician awareness and preferences, access to DXA scans and patient factors. There is a need for comprehensive and consistent guidance for both the initial and the long-term management of bone health in men with PC receiving ADT.

### Breast Cancer

Both the European Society for Medical Oncology (ESMO) and the American National Comprehensive Cancer Network (NCCN) recommend bone health assessment in women with iatrogenic premature ovarian failure and in patients treated with AIs for BC [70, 71]. The baseline evaluation should include a careful history to identify risk factors for fragility fractures [39, 72]. DXA assessment of BMD is recommended at baseline and should be repeated periodically, according to the local guidelines for osteoporosis management. The FRAX^®^ tool is also useful to estimate the 10-year risk of fracture [67]; however, the algorithm does not include cancer treatment as a risk factor. Further information may be provided by laboratory assessment including calcium, phosphate, vitamin D, parathyroid hormone (PTH) and creatinine clearance. Bone biomarkers (such as serum bone alkaline phosphatase and urinary *N*-telopeptide of type I collagen) have been investigated for their ability to predict osteoporosis and fragility fractures in BC [73], but their routine use in clinical practice is currently limited by the high inter- and intra-individual variability.

## Management of CTIBL

### CTIBL in Prostate Cancer

#### Lifestyle Measures

Both smoking and excessive alcohol intake are associated with reduced BMD and should be avoided [74]. Other consequences of ADT are sarcopenia and fatigue, both of which increase the likelihood of frailty, falls and fractures [75]. Regular exercise is helpful to minimise this risk, and supervised aerobic and resistance exercise programmes, performed at least twice a week for 12 weeks, are currently recommended for all men undergoing ADT [76–78].

#### Calcium and Vitamin D Supplementation

Men with PC are frequently deficient in both calcium and vitamin D [79]. Since vitamin D deficiency in men receiving ADT is independently associated with spinal fractures [80], supplementation with calcium and vitamin D should be considered in all men receiving ADT. However, the recommended doses (500–1000 mg calcium and 200–500 IU vitamin D per day) may be insufficient to prevent bone loss [81]. Further intervention studies to determine both the safety and efficacy of higher doses are required.

#### Bone-Targeted Agents (BTAs)

BTAs have an important role in the management of CTIBL. Bisphosphonates (BP) have high affinity for mineralised bone matrix, where they bind selectively to hydroxyapatite and are released during resorption. Ingestion of BP by osteoclasts results in their inhibition, either through induction of apoptosis (non-nitrogen-containing BP such as clodronate) or through inhibition of the mevalonate pathway required for osteoclastogenesis (nitrogen-containing BP such as zoledronate, ibandronate and pamidronate). Many BP are administered orally; however, the most comprehensively studied is zoledronate which is given intravenously. It requires dosage adjustment in patients with a creatinine clearance (CrCl) < 60 ml/min and is contraindicated in severe renal impairment (CrCl < 30 ml/min).

Denosumab is another BTA approved for the treatment of CTIBL. It is a fully humanised monoclonal IgG2 antibody that targets RANK-L and prevents its interaction with RANK on osteoclast precursors. Consequent inhibition of osteoclast differentiation and activation causes a rapid reduction in bone resorption [82].

Other antiresorptive drugs are currently widely used for the management of non-malignant bone disease, but are not specifically recommended in the CTIBL setting. These include calcitonin (the clinical investigation of which in cancer patients has been very limited) and teriparatide, a recombinant human PTH analogue. Teriparatide has been associated with an increased risk of osteosarcoma in patients receiving bone radiotherapy and is contraindicated for the treatment of CTIBL [83].

The potential role of SERM for the prevention of ADT-induced bone loss has been widely investigated by Smith and coworkers. In particular, 12-month treatment with either raloxifene [84] or toremifene [85] turned out to be capable to increase BMD at all sites, while reducing BTM levels, in men with non-metastatic PC undergoing ADT. When administered for 24 months, toremifene induced a 50% reduction in new vertebral fractures and significantly decreased the risk of fractures at all sites (*p* < 0.05 in both instances), as compared to placebo [86].

The role of denosumab in men with PC was evaluated in a randomised study of 1468 men receiving ADT for localised PC. Denosumab increased LS BMD by 5.6%, whereas those in the placebo group experienced a 1.0% loss (*p* < 0.001) [87]. Denosumab was also associated with a reduction in the incidence of new vertebral fractures after 36 months of treatment (1.5 vs. 3.9% with placebo; RR 0.38; 95% CI 0.19–0.78), and with a non-significant reduction in fractures at any site. Importantly, there were no between-group differences in adverse events.

Several studies have investigated the ability of BP to prevent CTIBL associated with ADT. BP have been found to increase LS and hip BMD when compared to placebo, and also to be associated with a significant reduction in BTM [88–92]. However, available evidence is limited by small patient numbers, heterogeneous populations, variations in the type and frequency of BP administration and different schedules of follow-up, all of which preclude effective meta-analysis. No study had sufficient power to detect differences in the incidence of fractures between groups. Only one non-randomised study of 94 patients attempted to compare the efficacy of different BP [93]. Large, prospective randomised studies of BP in men receiving ADT are necessary in order to determine the efficacy of BP for fracture prevention in this setting.

#### Guidelines for the Use of BTA in PC

**Fig. 1:**
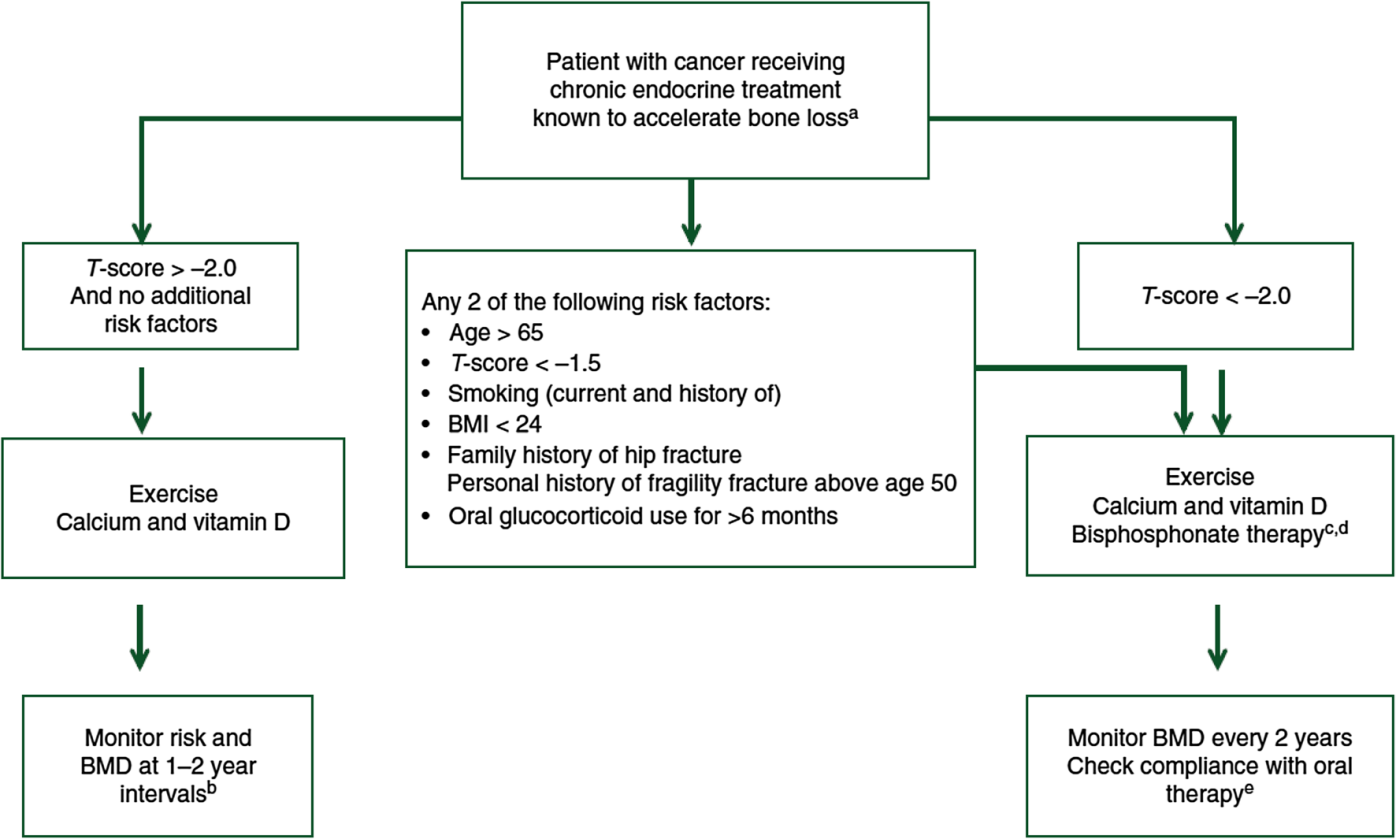
Algorithm for the assessment and management of cancer treatment-induced bone loss [70]. **a** Aromatase inhibitors and ovarian suppression therapy/oophorectomy for BC and androgen deprivation therapy for prostate cancer. **b** If patients experience an annual decrease in BMD of ≥ 10% (or ≥ 4–5% in patients who were osteopenic at baseline) using the same DXA machine, secondary causes of bone loss such as vitamin D deficiency should be evaluated and antiresorptive therapy initiated. Use lowest *t* score from spine and hip. **c** Six monthly intravenous zoledronate, weekly oral alendronate or risedronate or monthly oral ibandronate acceptable. **d** Denosumab may be a potential treatment option in some patients. **e** Although osteonecrosis of the jaw is a very rare event with bone protection doses of antiresorptives, regular dental care and attention to oral health are advisable. *BMD* bone mineral density, *BMI* body mass index, *DXA* dual-energy X-ray absorptiometry

European guidelines are available to guide the approach to CTIBL in PC patients [70]. These include a treatment algorithm for assessment and management of bone health (Fig. 1). Although a range of BP have been shown to prevent bone loss in men with locally advanced PC receiving ADT, denosumab is currently the only BTA that is licensed for the prevention of CTIBL in PC.

### CTIBL in BC

#### Lifestyle Measures

Limitation of alcohol consumption and smoking cessation are recommended. Moderate weight-bearing exercise should be practised regularly to take advantage of the beneficial effects of exercise on BMD [94].

#### Calcium and Vitamin D Supplementation

If dietary intake is inadequate, calcium supplementation is recommended (1000 mg/day) together with vitamin D supplementation (800–1000 IU/day). Concomitant steroid uptake interferes with vitamin D absorption and requires higher dosage [39, 95]. Elderly patients, and those with reduced sunlight exposure and/or physical activity, should be assessed for vitamin D serum levels and deficient levels treated with high-dose vitamin D followed by ongoing supplementation [94].

#### BTA

Several clinical trials have investigated the role of BP in the management of BC-associated CTIBL; the most important are summarised in Table 2. The earliest results came from small studies exploring the efficacy of oral clodronate. Postmenopausal women treated with adjuvant tamoxifen or toremifene were randomised to the clodronate or control group for 3 years, where clodronate was associated with a small BMD improvement at both LS (+ 1.0 vs. − 1.7%, *p* = 0.01) and FN (+ 2.4 vs. − 0.4%, *p* = 0.12), as compared to patients not receiving the BP [96].

**Table 2:** Clinical trials investigating the efficacy of bisphosphonates in preventing AI-induced CTIBL

Clinical trial	No. of patients	Follow-up period (months)	Experimental arm	Control arm	BMD variations at LS	References
ARIBON	131	24	Anastrozole + ibandronate	Anastrozole + placebo	+ 2.98 vs. − 3.22%	[98]
SABRE	234	24	Anastrozole + risedronate	Anastrozole + placebo	+ 2.2 vs. − 1.8%	[99]
IBIS II bone sub-study	1410	36	Anastrozole + risedronate	Anastrozole + placebo	+ 1.1 vs. − 2.6%	[100]
Z-FAST	602	61	Letrozole + upfront ZA	Letrozole + upfront ZA	+ 6.18–6.22 vs. − 2.42%	[101]
ZO-FAST	1065	60	Letrozole + upfront ZA	Letrozole + delayed ZA	+ 4.3 vs. − 5.4%	[102]
E-ZO-FAST	527	12	Letrozole + upfront ZA	Letrozole + delayed ZA	+ 2.72 vs. − 2.71%	[103]

*BMD* bone mineral density, *LS* lumbar spine, *ZA* zoledronic acid

The ARIBON trial reported that 2-year treatment with ibandronate induced a significant BMD gain at both LS and hip (+ 2.98 and + 0.60%, *p* < 0.01 for both) in osteopaenic postmenopausal women treated with adjuvant anastrozole [97]. Patients who continued ibandronate treatment up to 5 years underwent a mean lumbar BMD increase of + 3.19%, while those stopping the BP showed a BMD reduction at both LS (− 3.21%) and hip (− 5%) at the 5-year follow-up [98].

The efficacy of oral risedronate in postmenopausal women was investigated in both the SABRE trial and the IBIS II study. SABRE reported that those at moderate or high risk of fragility fractures significantly benefited from the addition of risedronate to adjuvant AI treatment [99]. IBIS II investigated the effect of anastrozole or placebo for BC prevention in women who were at high risk of BC. Those with osteopaenia or osteoporosis at baseline were included in a sub-study to assess bone protection with risedronate. After 3 years, the addition of risedronate to anastrozole was associated with a 1.1% (95% CI 0.2–2.1) increase in LS BMD compared to a 2.6% (95% CI − 4.0 to − 1.3) decrease with placebo (*p* < 0.0001) in osteopaenic women; among osteoporotic patients, those receiving placebo and risedronate experienced a greater gain in BMD at LS (3.9 vs. 1.2%, *p* = 0.006) and hip (1.5 vs. 0.3%, *p* = 0.12) than those treated with anastrozole with risedronate, but the BP still counterbalanced the AI-induced bone loss [100].

Zoledronate is the most extensively investigated intravenous BP in the CTIBL setting, both in pre- and postmenopausal patients. In the bone sub-protocol of the ABCSG-12 trial, 404 premenopausal women were randomised to receive hormone treatment (goserelin + anastrozole vs. goserelin + tamoxifen) with or without zoledronate. After 3 years, patients receiving the BP exhibited stable BMD at both LS and trochanter (+ 0.4 and + 0.8%, respectively), while at 60 months zoledronate treatment was associated with BMD increases at both sites, compared to baseline (4.0% at LS, *p* = 0.02; 3.9% at trochanter, *p* = 0.07) [51]. Those who received endocrine therapy alone experienced significant bone loss at both time-points, with the greatest loss being observed in the goserelin + anastrozole arm.

In postmenopausal women undergoing AI therapy, the Z-FAST, ZO-FAST and E-ZO-FAST trials compared upfront zoledronate treatment (4 mg every 6 months) with delayed administration, started after the detection of fractures or BMD decrease. At the final analyses, all these studies showed significantly higher BMD values in the “upfront” arms at LS, FN and hip, as compared to “delayed-treatment” groups [101–103]. In agreement with these data, Wagner-Johnston et al. described that upfront zoledronate addition to letrozole treatment, in postmenopausal patients previously treated with tamoxifen, was less frequently associated with LS BMD decrease as compared to delayed therapy. This difference was statistically significant after 5 years (*p* < 0.0001), while there were no significant differences in osteoporosis and fracture occurrence [104]. A recent study has also described a significant increase in LS BMD (+ 11.6%, *p* = 0.01) when zoledronate was given alongside adjuvant AI in women with pre-existing osteopaenia or osteoporosis [105].

Denosumab has also been investigated for its role in the prevention of AI-induced bone loss. In an initial randomised phase II study, 252 women with early HR + BC were randomised to receive an AI with or without denosumab (60 mg every 6 months for 2 years), and those in the denosumab arm experienced higher BMD gains at multiple sites, compared to controls [106]. The subsequent ABCSG-18 phase III trial prospectively evaluated the effects of adjuvant denosumab in postmenopausal patients with early HR + BC receiving AIs. A total of 3420 women were randomised to receive either denosumab or placebo every 6 months. Denosumab was associated with a significantly delayed time to first fracture and a reduced fracture incidence, compared to placebo (independently of baseline BMD) [107]. However, after denosumab discontinuation a rebound effect with accelerated bone loss was described [108, 109] leading to the proposal of a sequential BP administration to retain BMD values.

#### Guidelines for the Use of BTAs in BC

Current guidelines recommend adjuvant BTA treatment in those with a baseline *t* score < − 2 or at least two risk factors for fractures [110]. A panel of bone experts representing the major international societies that promote bone health and research (i.e. International Osteoporosis Foundation, IOF; Cancer and Bone Society, CABS; European Calcified Tissue Society, ECTS; International Expert Group for AIBL, IEG; European Society for Clinical and Economics Aspects of Osteoporosis, Osteoarthritis and Musculoskeletal Diseases, ESCEO; International Menopause Society, IMS) has recently published a position statement that recommends BTA administration also to patients with a baseline *t* score < − 1.5 with an additional risk factor for fracture, while those women with a baseline *t* score > − 2 and no fracture risk factors should be managed according to the BMD variations which occurred during the first year of AI treatment [94]. There is no consensus about the optimal treatment duration, but the Cancer Care Ontario (CCO) and American Society of Clinical Oncology (ASCO) guidelines do not recommend the administration of zoledronate for more than 5 years, while clodronate should be given for up to 3 years [111].

In premenopausal patients undergoing ovarian suppression, 4 mg zoledronate every 6 months is recommended in addition to calcium and vitamin D supplementation. In postmenopausal women, either oral or intravenous BP have proven efficacious in preventing bone loss during AI treatment, and thus the treatment choice should depend upon local guidelines, the different toxicity profiles and concurrent therapies [49, 70]. Further clinical trials are needed to compare different BP doses and schedules [111].

Compliance to oral BP should be regularly assessed and, if unsatisfactory, switching to an intravenous BTA should be considered [94].

A recent prospective study investigated adherence to CTIBL treatment guidelines by both patients and clinicians. Vitamin D and calcium supplementation was prescribed in three quarters of those receiving AIs, but failure to comply with guidelines was identified in 11.6% of cases. A total of 12% (54 of 438 patients) received a BP, but seven patients who were osteoporotic did not receive antiresorptive medication [112]. These data underline the need for clarification and improved awareness of current guidelines.

There is still a debate about the administration of denosumab in the adjuvant setting; European bone experts suggest that it might be offered at the same dosage administered to patients with osteoporosis [49], while US clinicians underline the need for more consistent data, before making any recommendation [111].

Recent evidence suggests that adjuvant BP also reduce bone recurrence and improve survival in postmenopausal patients with BC [113]. Although the routine use of BP in this setting has not received regulatory approval, bone experts and oncologists recommend their administration to postmenopausal women with intermediate/high risk of BC (T2-4, N1-3, grade 2–3, ER negative or Her2 positive), regardless of BMD values [110, 111].

### BTA Safety

BTAs are generally well tolerated, especially at the dosage recommended for CTIBL prevention [70].

Intravenous BP, such as zoledronate, seem to be more frequently associated with acute phase reactions than the oral ones. Symptoms include transient bone pain, arthralgia or myalgia, fever and nausea [111]. On the other hand, oral BP can cause esophagitis and other gastrointestinal disorders; for this reason, it is advised to take them on an empty stomach and remain upright for at least 30 min [111].

Hypocalcaemia is more likely to occur during denosumab treatment but can easily be prevented by the regular monitoring of serum calcium levels and the concomitant administration of calcium and vitamin D supplements [70].

Osteonecrosis of the jaw (ONJ) is one of the most important adverse events occurring during BTA treatment. However, it is more common (incidence of approximately 1.3%) in bone metastatic patients who receive BTAs on a monthly basis, rather than those undergoing less intensive treatment for osteoporosis or CTIBL management [49].

Nevertheless, a careful dental examination and appropriate preventive dentistry are recommended before BTA administration, and patients should be advised to maintain good oral hygiene. If possible, invasive dental procedures, such as tooth extractions and implants, should be avoided during BTA treatment [49, 70].

## Conclusion

The improved survival of patients with BC and PC has led to increasing awareness of survivorship issues, including the long-term consequences of cancer treatment. Current treatments for both BC and PC adversely affect bone health via several mechanisms, increasing the risk of osteoporosis and fracture. These potentially devastating complications may be avoided by lifestyle changes and the use of BTAs, with zoledronate and denosumab being the most comprehensively studied agents. Zoledronate has been shown to prevent the loss of BMD in women with BC who experience premature menopause, in postmenopausal women receiving an AI and in men with PC undergoing ADT. However, these improvements have unfortunately not been translated into a clear reduction in fracture incidence. Denosumab is licensed for the prevention of CTIBL due to its proven ability to reduce the incidence of fractures and has become the treatment of choice for patients at high risk of fracture. Recently published guidelines are available to guide the assessment and treatment of CTIBL in both PC and BC. There is a need to improve awareness of CTIBL amongst clinicians and members of the multidisciplinary team, in order to ensure that these are widely and consistently implemented in clinical practice and that patients receive the best possible treatment.
